# Geriatric Choosing Wisely choice of recommendations in France: a pragmatic approach based on clinical audits

**DOI:** 10.1186/s12877-021-02619-7

**Published:** 2021-12-15

**Authors:** T. Tannou, E. Menand, D. Veillard, J. Berthou Contreras, C. Slekovec, V. Daucourt, D. Somme, A. Corvol, on behalf of French Choosing Wisely Geriatric Group of the SFGG, T. Tannou, E. Menand, D. Veillard, D. Somme, A. Corvol

**Affiliations:** 1 Centre Hospitalier et Universitaire de Besançon, Service de Gériatrie, F-25000 Besançon, France; 2 Centre Hospitalier et Universitaire de Besançon, INSERM CIC 1431, équipe “Ethique et progrès médical”, F-25000 Besançon, France; 3 Université de Franche-Comté, UFR des Sciences de la Santé, Laboratoire de Recherches Intégratives en Neurosciences et Psychologie Cognitive, F-25000 Besançon, France; 4 Centre de recherche, Institut Universitaire de Gériatrie, Montréal, QC Canada; 5 Service de gériatrie, CHU de Besançon, Boulevard Fleming, 25030 Besancon, France; 6 Univ Rennes, CHU Rennes, Service de Gériatrie, F-35000 Rennes, France; 7 CAPPS, structure régionale d’appui à la qualité des soins et la sécurité des patients, Rennes, France; 8 Univ Rennes, CHU Rennes, Service de Santé Publique, F-35000 Rennes, France; 9 OMéDIT, Observatoire du Médicament des Dispositifs médicaux et des Innovations Thérapeutiques, CHU de Besançon, Besançon, France; 10 CPIAS, Centre d’appui pour la Prévention des Infections Associées aux Soins Bourgogne-Franche-Comté, CHU de Besançon, Besançon, France; 11 RéQua, Structure régionale d’appui à la qualité des soins et la sécurité des patients, Besançon, France; 12 Univ Rennes, CHU Rennes, CNRS, ARENES, UMR 6051, F-35000 Rennes, France

**Keywords:** Choosing wisely, Clinical audit, Geriatrics, Deprescribing

## Abstract

**Background:**

The international Choosing Wisely campaign seeks to improve the appropriateness of care, notably through large campaigns among physicians and users designed to raise awareness of the risks inherent in overmedication.

**Methods:**

In deploying the Choosing Wisely campaign, the French Society of Geriatrics and Gerontology chose early operationalization via a tool for clinical audit over a limited area before progressive dissemination. This enabled validation of four consensual recommendations concerning the management of urinary tract infections, the prolonged use of anxiolytics, the use of neuroleptics in dementia syndromes, and the use of statins in primary prevention. The fifth recommendation concerns the importance of a dialogue on the level of care. It was written by patient representatives directly involved in the campaign.

**Results:**

The first cross-regional campaign in France involved 5337 chart screenings in 43 health facilities. Analysis of the results showed an important variability in practices between institutions and significant percentage of inappropriate prescriptions, notably of psychotropic medication.

**Discussion:**

The high rate of participation of target institutions shows that geriatrics professionals are interested in the evaluation and optimization of professional practices. Frequent overuse of psychotropic medication highlights the need of campaigns to raise awareness and encourage deprescribing.

## Background

In 2012, the American Board of Internal Medicine Foundation [1] launched the international Choosing Wisely™ campaign, with the aim of improving the appropriateness of treatments, notably through wide-ranging initiatives designed to raise awareness among physicians and users of the risks inherent in overmedication [2]. Targeting both prescribers and users, this initiative placed patients at the centre of health care decision making by providing them with the information needed to make informed decisions. In practice, the participating learned societies draw up a list of 5 prescriptions (for treatments or additional exams) commonly used in the speciality concerned and for which the risk of inappropriate prescription is high and well documented [3]. Today, Choosing Wisely exists in over 20 countries and involves several dozen learned societies [4].

In 2015, the Fédération Hospitalière de France (French public hospital federation) signed the charter of commitment to the Choosing Wisely™ campaign. The French Society of Geriatrics and Gerontology (SFGG) join early this initiative by proposing a list of recommendations likely, through assessment of adherence to recommendations and correction of any deviations, to improve the appropriateness of care.

There is much at stake in this approach in geriatrics. Older persons are particularly exposed to the risk of prescriptions of drugs and of additional inappropriate tests. Indeed, polypharmacy, which is frequent in the older patients, increases the risk of inappropriate prescription and of side effects, which are more frequent and serious in this population with multimorbidity [5, 6]. This multimorbidity leads to atypical disease presentations [7] which can result in the use of additional tests in a clinical context that differs from that in which the tests were validated. This leads to a high risk of inappropriate decisions taken on the basis of the test results. In parallel, this has a negative impact on both the patients’ quality of life and the health system. The person-centred approach is particularly important given that care objectives may vary greatly from one patient to another [8]. Shared decision making is hard to promote if the patient suffers from sensory or cognitive impairments and if, as is often the case, family carers are involved in decision making [9]. Choosing Wisely™ seeks to give the practitioner and the patient tools that promote dialogue on the appropriateness of the prescriptions envisaged.

In deploying the Choosing Wisely™ approach, the SFGG chose a method and implementation based on iterative co-construction, with the drawing up of recommendations, followed by 2 regional waves of testing with, each time, analysis of the results to enable adaptation of the recommendations, leading to the national campaign. These regional waves of testing and their interim results leading to the national wave of testing are presented in “Methodology,” and the findings of the 2019 national campaign are given in “Results.”

## Methods

**Fig. 1:**
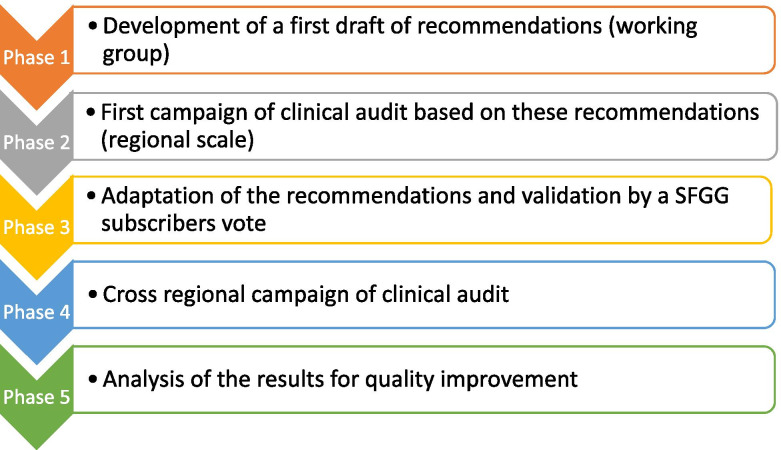
General methodology of the French Choosing Wisely approach

Our approach to the implementation of a Choosing Wisely campaign in France is based on a 5-phase process, presented in Fig. 1. These phases include the initial development of recommendations, their local implementation, and then the validation of recommendations for a national campaign. These 3 phases are presented in the methods section. The results of the national survey are presented in the results section, followed by the analysis and opportunities for improvement, in discussion section.

### The choice of recommendations: a pragmatic approach

In 2016, the SFGG decided to draw up a first list of recommendations. For this, it mobilized a first working group comprising university and non-university geriatricians, an SFGG representative, and a representative of users. The SFGG also sought methodological help from the support team for quality and security of care in the French region of Brittany. The working group based its “top five list” on the recommendations for Choosing Wisely™ campaigns published by American and Canadian learned societies, a literature review, and the experiences of practitioners and users concerning the frequency of certain inappropriate prescriptions in France. The working group prepared a list of 10 proposals, which a panel of 15 geriatricians working in five different institutions then classified in order of importance according to their wish to see them included in a communication campaign. Five recommendations were selected, relating to screening for and treatment of bacterial urinary tract infections, the prescription of antipsychotic medication in dementia syndrome, the prolonged use of anxiolytics, the re-assessment of chronic treatments, and enteral nutrition in major neurocognitive disorders (Table 1).

**Table 1:** SFGG Choosing Wisely recommendations. Five recommendations were validated. Three were not selected following campaigns to evaluate professional practices and voting at the annual SFGG meeting

SFGG Choosing Wisely Recommendations	
Recommendations validated after the 2016 evaluation of professional practices campaign and the SFGG vote	
Asymptomatic urinary tract infections should be neither screened for nor treated.	
There is no indication for prolonged treatment with anxiolytics. In the case of ongoing treatment, the value of weaning and the means to achieve it should be explained to the patient.	
In the case of behavioural disorders in a patient with dementia syndrome (major neurocognitive disorder), neuroleptics should only be prescribed as a last resort in the event of the failure of non-pharmaceutical measures, for a short period, after analysis of the causes.	
Recommendations not chosen after the 2016 evaluation of professional practices campaign	
*The treatments of frail older adults should be regularly reviewed in light of their expected benefit, the potential risks, and, for preventive treatments, life expectancy.*	
*There is no need to propose enteral nutrition in swallowing disorders that occur in a context of cognitive disorders that arise secondary to a neurodegenerative disease.*	
Recommendation validated after the 2019 evaluation of professional practices campaign and the SFGG vote	
There is no indication to prescribe or to continue statin treatment in people aged over 80 who have never presented cardiovascular incidents (primary prevention).	
Recommendation not selected after the SFGG vote	
*There is no indication to prescribe or to continue antiplatelet agents in primary prevention in non-diabetics over 75 years of age.*	
Recommendation by users	
Starting from the first days of admission to hospital or to a care home, there should be a dialogue with the patient (and, if he/she wishes, with family carers) on the nature of the care to be implemented, so that the care is based on the patient’s needs and expectations.	

### Conduct of the first wave of evaluations of adherence to recommendations

Early operationalization of these recommendations was promoted, in the form of clinical audits [10]. The aim for this stage of testing was to check the feasibility of this approach in the French context, and notably the adherence of clinicians to each recommendation. The participating teams could choose which recommendations they wanted to use to assess their practices. This initiative was followed in acute, post-acute and long-term care departments, in the form of a retrospective survey on a given day and over a pilot region, Brittany. The quality managers and the medical commission of the hospitals of this region were asked by post to participate in this test campaign. In parallel, the regional geriatrics society mobilized geriatricians, and regional pharmacy societies have mobilised hospital pharmacists. This wave was coordinated by the Breton support team for quality and security of care, which developed an online data collection tool. Each team was invited to evaluate the medical charts of 30 patients hospitalized on a given day, for each recommendation that the team chose to assess. Following data collection, these teams received personalized feedback on their results, recommendation by recommendation.

### Results and adaptation of the methodology after the first wave

There was strong uptake by professionals as 27 departments in 16 different institutions took part in two waves of clinical audit, in 2016 and 2017. In all, 1060 patients of average age 86 were included. Among 981 patients included for the recommendation for screening for asymptomatic bacterial urinary tract infections, urine test strips had been used in 132 in the previous 3 days, 67 (7%) of them in the absence of symptoms. Of 353 patients included for the recommendation on anxiolytic treatments, 184 (50%) received one of the target treatments. Of the 707 patients included for the recommendation on antipsychotics, 138 (20%) received one of the target treatments, including 106 (15%) whereas they presented dementia syndrome.

The results and the participations in this first wave were analyzed so as to check whether the recommendations were aligned with the quality criteria of the Choosing Wisely™ campaign, notably: consensus, frequency of inappropriateness, possibility of reducing this inappropriateness.

Following analysis of the findings, the project team decided to remove two recommendations from the top five list. The first, on the systematic re-evaluation of preventive treatments as a function of life expectancy, proved too vague to be an effective tool for clinical audit. The second, on feeding via a nasogastric tube in swallowing disorders associated with major neurocognitive disorders, involved too few patients for the departments concerned and so for the teams did not correspond to sufficiently common practices. Three of the five recommendations initially proposed were therefore selected after this first campaign. However, the evaluation charts were optimized to facilitate interpretation of the results. For example, we extended the period considered for urine dipsticks from 3 to 7 days and changed the order of the questions on neuroleptics, to get the ratio of prescription among patients with dementia.

In the framework of the involvement of patients according to the principles of Choosing Wisely™ campaigns [11], the project group proposed, on the one hand, that users be associated with the review of proposals in order to harmonize the vocabulary and promote understanding and acceptance of the proposals by non-professionals, and, on the other hand, that users directly propose one of the recommendations. Mobilized in the context of the working group, users wanted this recommendation to relate to the importance of the dialogue established with the patient (and his or her family carers) on the nature of his or her care. A specific proposal on this theme by the users was therefore written by a group of patients supported by a physician from the regional support team for quality of care. As this proposal was prioritized by the user group, it was decided not to submit it to geriatricians for validation to support user involvement.

Concerning the proposal to re-evaluate chronic treatments, given the risks of not meeting the quality criteria of the recommendations, it was decided to target single treatments. The two targeted treatments proposed by the working group were antiplatelet drugs and statins in primary prevention (Table 1).

### Validation of the recommendations with a view to a national campaign

To validate the choice of recommendations and to initiate a national campaign, a vote was taken at the 2018 annual meeting of the SFGG on the working group’s five proposals, four of which were selected, along with that of the users. The proposals concerning urinary tract infections, statins, anxiolytics, and neuroleptics were validated, deemed priorities or highly relevant by more than 70% of the 508 voters, with respectively 375 (74%), 362 (71%), 389 (77%), and 420 (83%) votes. The disagreement rate was less than 3% (12 voters for urinary tract infections, 6 for statins, 3 for anxiolytics, and 2 for neuroleptics). The proposal concerning the use of antiplatelet drugs was not selected, as it appeared less consensual: 21 voters (4%) disagreed with it, 40 voters (8%) did not consider it appropriate for inclusion in the campaign, and 332 (65%) considered it a priority or highly relevant. Table 1 presents the five recommendations finally selected.

### The 2019 cross-regional survey: setting and methods

Validation of these SFGG recommendations allowed the roll-out of a first Choosing Wisely™ cross-regional campaign in geriatrics, in France. It was conducted in two French regions through the involvement of university geriatricians and user representatives, backed by three regional support teams, i.e. teams for quality of care, prevention of healthcare-associated infection and good use of drugs.

The methodology used was that tested before in the first campaign: regional, retrospective survey on a given day, based on a clinical audit validated for each recommendation. The health facilities were informed by letters addressed to their directors and to their medical commission presidents, and to the quality managers, pharmacists, and hygienists. In parallel, regional geriatrics societies mobilized the geriatricians of these institutions. Data on each of the four medical proposals were collected in a three-part questionnaire: the first part was used to validate the inclusion criteria; the second part explored the appropriateness of patient care with regard to the recommendation; and the third part examined the justifications for inappropriateness with a view to proposing avenues for improvement. The data were collected online from the medical charts. Each team chose the recommendations it wanted to use to evaluate its practices. Each participating facility had to include at least 30 patients aged 75 or older for each of the selected recommendations (between 1 and 4). Proposal 5, on the quality of the dialogue between the physician and the patient, has not been included in that survey as it could not be evaluated by chart audit. If the department had fewer than 30 older adults, all of them were included. If the department had more than 30 older adults, 30 consecutive beds were selected for inclusion. The survey day was randomly determined in each institution by the evaluator.

After the survey, the results were sent to each institution and a regional and cross-regional comparison was organized, in order to shed light on the overall situation and, from key points identified in the institutions, to propose personalized support in association with the regional support groups.

The statistical analysis was done on absolute data, but also proportionally compared with the whole data set. Quantitative variables were defined by their means and standard deviations, and qualitative variables by their number and proportion.

### Results of the 2019 cross-regional campaign

The survey was conducted between March and September 2019 in 43 care facilities, and concerned 17 acute care departments, 25 post-acute care departments, and 39 long-term care departments.

**Fig. 2:**
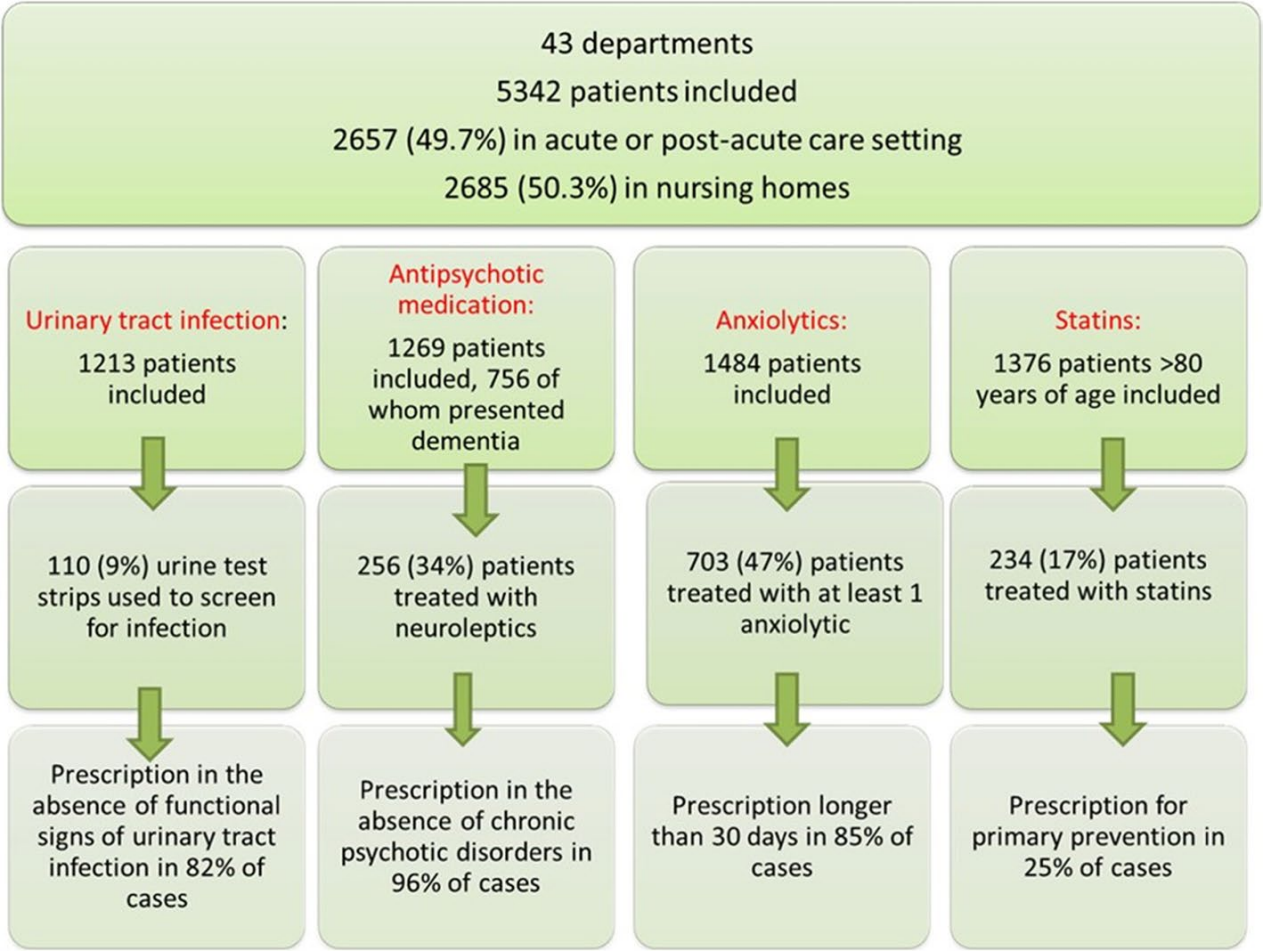
Summary of the main results, with the number of data collected per item, and the deviations from the Choosing Wisely™ recommendations

The campaign comprised 5342 analyses of adherence to recommendations, half of them in nursing homes and the other half in acute and post-acute care departments (Fig. 2). The recommendation accounting for the greatest number of analyses was that concerning benzodiazepines, followed in terms of the medical charts assessed by statins, antipsychotic medication and, lastly, urinary tract infections. Some differences were observed depending on the type of structure. Detailed results are provided in Table 2.

**Table 2:** Results regarding Recommendation 1 according to the type of structure

	Acute/post-acute	Nursing Home
**Recommendation 1**	592 screening	621 screening
	93 urine tests within the previous 7 days	17 urine tests within the previous 7 days
	15 urine tests associated with clinical symptoms (17%)	5 urine tests associated with clinical symptoms (29%)
	78 urine tests without specific clinical symptoms or surgical procedure (83%)	12 urine tests without specific clinical symptoms or surgical procedure (71%)

### Results for recommendation 1: asymptomatic urinary tract infections should be neither screened for nor treated

Screening for urinary tract infections by means of urine tests strips and/or urine culture was sought in the medical charts of 1213 patients over 75 years of age, 592 of them in acute or post-acute care and 621 in nursing homes, across 35 different administrative divisions or departments. Of these, 110 (9%) had in the previous 7 days undergone urine sampling, outside a specific interventional protocol or urinary catheterization (Table 2). In this group, 20 (18%) patients presented functional signs of irritation and/or obstruction. Ninety patients without specific symptoms or signs therefore underwent screening or urine culture, which represents 82% of prescriptions (83% in acute and post-acute departments, 71% in nursing homes). The reasons provided by the clinicians were mainly the presence of psychological-behavioural disorders or signs of sepsis without an obvious entry point, and screening, notably by systematic use of urine test strips upon admission to the department. This practice of systematic screening in acute care settings led to substantial variability between institutions. Thus, some facilities, particularly in post-acute care, require systematic urine screening on admission. This has a significant impact on the results. For example, of the 96 tests administered in acute or post-acute facilities, 26 were administered in a single facility, 24 of them without clinical justification.

### Results for recommendation 2: in a person with dementia syndrome presenting behavioural disorders, neuroleptics should only be prescribed as a last resort

The use of neuroleptics in patients aged over 75 who presented a major neurocognitive disorder was investigated in 1269 patients, 563 in acute or post-acute care and 706 in nursing homes, in 41 different departments. Of 756 patients with dementia syndrome, 256 (34%) had at least one prescription for neuroleptics on the day of the survey (38% in nursing homes and 25% in acute or post-acute care departments) Details are provided in Table 3. The main reason reported by the clinicians for continuing to use neuroleptics was extension of a previous prescription without re-evaluation upon admission of the patient.

**Table 3:** Results regarding Recommendation 2 according to the type of structure

	Acute/post-acute	Nursing Home
**Recommendation 2**	563 screening	706 screening
	238 older adults living with dementia	518 older adults living with dementia
	59 older adults living with dementia under neuroleptics (25%)	197 older adults living with dementia under neuroleptics (38%)
	4 had a weaning attempt to stop the prescription	32 had a weaning attempt to stop the prescription

### Results for recommendation 3: there is no indication for prolonged anxiolytic treatment

The use of anxiolytics for more than 30 days was investigated in 1484 over-75 s, 887 of whom were in acute or post-acute departments and 597 in nursing homes, in 48 different departments. Anxiolytic treatment was used in 703 patients, that is in 40% of care home residents and in 52% of hospitalized patients. This prescription had lasted more than 30 days in 600 of these over-75 s, that is in 88% of nursing home residents and in 84% of hospitalized patients (Table 4). Among these cases, only 24 treatments (i.e. 4%) corresponded to the criteria of appropriateness, in other words the patient was involved in weaning and was informed about its value and how to achieve it. In over 80% of cases, the clinician conducting the survey justified prescription beyond 4 weeks by the persistence of manifestations of anxiety and/or sleep disorders. No clear indication was retrieved in the charts for 119 prescriptions (17%).

**Table 4:** Results regarding Recommendation 3 according to the type of structure

	Acute/post-acute	Nursing Home
**Recommendation 3**	887 screening	597 screening
	463 older adults were on benzodiazepine (52%)	240 older adults were on benzodiazepine (40%)
	Among them, 75 older adults on benzodiazepine for < 30 days (16%), and 388 older adults were on benzodiazepine for > 30 days (84%)	Among them, 28 older adults on benzodiazepine for < 30 days (12%), and 212 older adults were on benzodiazepine for > 30 days (88%)
	19 were offered benzodiazepine weaning and/or withdrawal support	5 were offered benzodiazepine weaning and/or withdrawal support

### Results for recommendation 4: there is no indication to prescribe or continue statin treatment in a patient over 80 years of age who has never presented cardiovascular incidents

Prescription of statins to patients aged 80 or more was evaluated for 1376 patients, of whom 615 were in acute or post-acute departments and 761 in nursing homes, in 37 different departments. Among these, 230 patients were taking statins at the time of the survey, i.e. 26% of hospitalized patients and 11% of care home residents (Table 5). One-quarter of these patients taking statins were doing so for primary prevention (30% in acute or post-acute departments and 13% in nursing homes). The clinicians justified this prescription by the patient’s high cardiovascular risk (at least 2 cardiovascular risk factors, associated with diabetes in half of the cases).

**Table 5:** Results regarding Recommendation 4 according to the type of structure

	Acute/post-acute	Nursing Home
**Recommendation 4**	615 screening among patients > 80	761 screening among patients > 80
	158 adults > 80 years old on statins (26%)	78 adults > 80 years old on statins (10%)
	47 of them were on statin for primary prevention (30%)	10 of them were on statin for primary prevention (13%)
	8 medical records mentioned attempted withdrawal	0 medical records mentioned attempted withdrawal

## Discussion

### Analysis and specificities of the results

With antibiotic therapy of urinary tract infections, one of the key points revealed by our study is the role of systematic screening, in particular through medical tests at admission. Although the approach to urinary symptoms is particularly complex in dependent older adults [12], a reasoned approach to antibiotics, especially in nursing homes, is essential and must be based on the raising of awareness among health care personnel [13, 14].

Our findings confirm the widespread overprescribing of psychotropic medication already well documented in the international literature [15]. A feature specific to France relates to the frequency of prolonged use of benzodiazepines in older patients [16], which explains the French Choosing Wisely™ recommendation to curtail this treatment, whereas the Choosing Wisely™ recommendations of American, Canadian, Australian, German, and Italian geriatrics societies relate to not introducing this treatment first line.

One-third of older patients with dementia in our sample were treated with neuroleptics for behavioural disorders, whereas this treatment is indicated only as a last resort. This proportion is high, but below that observed, for example, in long-term care centres in Canada, where the prescription concerned more than half of the residents with dementia [17]. Deprescribing psychotropic drugs for older persons is problematic, but possible. Several programs, in particular in long-stay settings, based on the joint involvement of patients, carers, prescribers, and pharmacists using non-pharmaceutical interventions have proven effective [18].

The prevalence of statin prescription that we found is below that usually noted in populations of the same age [19], which may show that the geriatricians targeted by our campaign were receptive to deprescribing preventive cardiovascular medication in at-risk populations [20]. Furthermore, the populations targeted by our approach mostly have a limited life expectancy.

The results of these clinical audits show, on the one hand, great variability in practices between institutions, and, on the other hand, a significant percentage of inappropriate prescriptions for all recommendations, which empirically validates the choice of targeted items. It is worth recalling that the Choosing Wisely™ campaign targets prescriptions at high risk of being inappropriate with regard to good practice recommendations or to scientific data on the subject. The aim of the campaign is not to prohibit certain prescriptions, but to raise awareness among practitioners and patients that careful thought should be given to such prescriptions. There may be patients for whom clinicians choose, in light of specific clinical features or because of the patient’s medical history or situation, to prescribe benzodiazepines long term or to treat a urinary infection in the absence of obvious functional signs. Nevertheless, our data indicate a frequency of such choices that does not fully accord with good clinical practice.

### Specificities of the choosing wisely™ approach in geriatrics in France

The first French national Choosing Wisely™ campaign in geriatrics was conducted in accordance with the dynamics and philosophy of the worldwide campaign, and had two specific features.

First, there was early roll-out of campaigns to evaluate professional practices. This fast operationalization enabled us to check the feasibility of the approach and the adherence of the care professionals. The validation by vote at the annual SFGG meeting legitimized the working group’s extension of these campaigns across regions and enabled wide-ranging communication with the professionals. Many learned societies chose a consensus by the Delphi method to draw up their “list of 5”, or chose an online survey of their members [2, 21, 22]. The SFGG approach allowed us to involve hands-on practitioners, in addition to learned society members, and to check their motives for adopting the tools proposed with a view to implementation. The participants were enlisted by voting and by the three waves of clinical audit not as recipients of messages, but rather as implementers of their own choices.

Second, there was the role of clinicians. In formulating proposals and arguments, a partnership was instigated by choosing a text for both professionals and users, who could choose and draft recommendations.

However, this approach has its limitations. Patient chart audits give only an indirect picture of practices. Some prescriptions may be justified, despite not following the recommendations. The objective of such campaign is mainly to bring to light potentially inappropriate prescriptions. Patients included may not be representative, since participation of health facilities was on a voluntary basis.

### Perspectives of the French initiative

This first experience mobilized many stakeholders, in both the hospital and medical-social sectors, to review their professional practices. This was made possible by the mobilization of clinicians, in particular university geriatricians, who have taken the lead in this campaign, and of support groups that have made available their organizational know-how for data collection and analysis.

This approach included feedback of the results to the participating institutions, each of which was therefore able to compare its results with those of other institutions. The findings from studies in other countries show that this initiative based on numerical results is a powerful means of changing practices [4]. However, we think that simple sharing of the results will not be enough to change practices. We need to develop new tools, such as decision-making aids, and to roll out large-scale initiatives in all medical specialities to explain that “more is not better” and to complete the prescriber-centred approach [2]. The organizations involved in the Choosing Wisely™ campaign in France, along with other clinicians, pharmacists, medical technologists, and user associations, should be able to participate in a multiprofessional roll-out of training courses and support that target these issues, in line with international deprescribing initiatives.

## Conclusion

The Choosing Wisely™ initiative is based on the concept that appropriateness of care is a condition for its quality and is related to patient centered goals of care. Our original model is based on a progressive implementation, locally and then nationally, of recommendations tested and validated under the aegis of the French Society of Geriatrics and Gerontology. The campaigns confirmed the choices of the recommendations and underscored the physicians’ commitment to best prescribing practices. The next steps are the organization of regular national campaigns, including an evaluation of the fifth recommendation on dialogue, by direct interview of the patients. Further research will be necessary to specify the tools and support needed to make significant progress in practice. The frequent overuse of psychotropic drugs underlines the need for awareness campaigns and incentives for deprescribing.

## Data Availability

The datasets generated and analysed during the current study are available from the corresponding author on reasonable request.
